# Protective Effect of Yang Mi Ryung® Extract on Noise-Induced Hearing Loss in Mice

**DOI:** 10.1155/2017/9814836

**Published:** 2017-11-15

**Authors:** Min Soo Kim, SeongAe Kwak, Heumyoung Baek, Zewu Li, Seong-Kyu Choe, Kyung Song

**Affiliations:** ^1^Center for Metabolic Function Regulation and Department of Microbiology, School of Medicine, Wonkwang University, Iksan 54538, Republic of Korea; ^2^Zoonosis Research Center, School of Medicine, Wonkwang University, Iksan, Jeonbuk 54538, Republic of Korea; ^3^Jeongwoo Pharmaceutical Co., Ltd., Asan, Chungnam 336885, Republic of Korea; ^4^Professional Graduate School of Oriental Medicine, Wonkwang University, Iksan, Jeonbuk 54538, Republic of Korea; ^5^Integrated Omics Institute, Wonkwang University, Iksan, Jeonbuk 54538, Republic of Korea; ^6^Department of Pharmacy, College of Pharmacy, Wonkwang University, Iksan, Jeonbuk 54538, Republic of Korea; ^7^Institute of Pharmaceutical Research and Development, Wonkwang University, Iksan, Jeonbuk 54538, Republic of Korea

## Abstract

Noise-induced hearing loss (NIHL) results from the damage of the delicate hair cells inside the ear after excessive stimulation of noise. Unlike certain lower animals such as amphibians, fishes, and birds, in humans, hair cells cannot be regenerated once they are killed or damaged; thus, there are no therapeutic options to cure NIHL. Therefore, it is more important to protect hair cells from the noise before the damage occurs. In this study, we report the protective effect of Yang Mi Ryung extract (YMRE) against NIHL; this novel therapeutic property of YMRE has not been reported previously. Our data demonstrates that the hearing ability damaged by noise is markedly restored in mice preadministrated with YMRE before noise exposure, to the level of normal control group. Our study also provides the molecular mechanism underlying the protective effect of YMRE against NIHL by showing that YMRE significantly blocks noise-induced apoptotic cell death and reduces reactive oxygen species (ROS) production in cochleae. Moreover, quantitative polymerase chain reaction (qPCR) analysis demonstrates that YMRE has anti-inflammatory properties, suppressing the mRNA levels of TNF*α* and IL-1*β* induced by noise exposure. In conclusion, YMRE could be a useful preventive intervention to prevent hearing impairment induced by the exposure to excessive noise.

## 1. Introduction

Loud noises such as firearms, explosions, sirens, televisions, radios, and noise from heavy city traffic are known to be the common causes of hearing loss [1]. Even though short exposure to loud noise may cause only temporary hearing loss that could disappear within a few hours or days, excessive noise exposure and long-lasting loud noise may result in permanent or gradual damage to hearing. In addition to environmental noise, recreational activities including listening to portable music devices at high volume through headsets, hunting, and attending loud concerts could increase the risk for noise-related hearing loss. Although those sounds at safe levels do not damage our ability to hear, the long, continuous, or repeated exposure to loud or intensive sounds can damage sensitive structures in the inner ear. Mechanistically, the intense metabolic activity induced by noise stimulation may make hair cells undergo oxidative stress, resulting in the cell death leading to noise-induced hearing loss (NIHL). In addition, blood flow in the cochlea has been well known as an critical factor for hearing function, and vasoconstriction induced by noise clearly contributes to NIHL [2–4].

Accumulating evidence has demonstrated that the mechanical damage and biochemical signaling pathways inducing apoptotic cell death are two main signaling pathways causing cochleae damage following noise stimulation. It has been reported that activation of apoptotic markers and TNF receptors, as well as morphological changes, occurred in animal models after noise. One of the pathways known to trigger apoptotic cell death in NIHL involves reactive oxygen species (ROS) and similar reactive species [4, 5]. A number of studies revealed that an increased level of ROS was commonly detected following noise exposure, leading to the cellular responses causing tissue injury [6–8]. Such damage by ROS is mediated by several oxidative stress-responsive signaling pathways and proinflammatory pathways including NF-*κ*B, JAK2/STAT3, JNK, IL-16, and IL-1, suggesting that those signaling molecules may be potential targets in protecting cells from noise-induced damage [9–11]. Moreover, ROS is known to deplete the intracochlear antioxidants and inhibit antioxidant enzymes including glutathione peroxidase [12], superoxide dismutases (SOD) [13, 14], NQO1, catalase, and hemeoxygenase-1 (HO-1) [15]. Such antioxidant enzymes are key players in the defense system that living cells have developed to protect the hair cells against the destructive effects of ROS and have been targets for ototoxic stimuli including noise [16].

Yang Mi Ryung (YMR, Jeongwoo Pharmaceutical Co., Ltd) is over-the-counter (OTC) medicine approved by the Korean Pharmaceutical Food and Drug Administration (KFDA) and herbal medicine whose composition originated and was altered from Jogyeongjongok-Tang included in Gogeumuigam, a Chinese book about traditional medicine. The composition of YMR consists of 16 herbal medicines, and there are a variety of OTC drugs produced by many pharmaceutical companies using the composition of YMR and formulated into pills or granules. YMR is known to improve blood flow and has primarily been used in the field of oriental medicine for the treatment of circulatory disorders such as female infertility, menstrual disorders as well as leucorrhea, menstrual cramping, urinary frequency, and vomiting.

In this study, we tested whether YMR, a useful medicine to improve blood flow, had a protective effect on NIHL in an animal model. Using the YMR extract (YMRE), the effect of YMR on NIHL was analyzed on the axis of ROS pathway and inflammation.

## 2. Materials and Methods

### 2.1. Animals and Overall Design

Twenty to 36 healthy Balb/C male mice (weight: 21 ± 1 g, 8 weeks old) were used, and their hearing ability was confirmed to be within normal range by auditory brainstem response (ABR) measurements. Animals were randomly divided into two groups—control mice (0.9% saline solution, PO) and mice administered with YMRE (100 mg/kg/day, PO) alone. Each group of mice was further divided into two subgroups after 5 weeks of YMRE treatment or normal saline. Mice in one subgroup of each group were then exposed to 100 dB noise for 3 h to induce hearing loss: subdivided groups included control mice with or without noise (0.9% saline solution) and YMRE (100  mg/kg/day, PO) group with or without noise, hereinafter referred to in this study as Cont, noise, YMRE, and YMRE + noise, respectively. This study was reviewed by the Committee for Ethics in Animal Experiments of the Wonkwang University (WKU16-63) and carried out under Korean law and the Guidelines for Animal Experiments.

### 2.2. Preparation and Yield of YMRE (The Extract of YMR)

Yang Mi Ryung extract (YMRE) used in this study was kindly provided by Jeongwoo Pharmaceutical Company, and tests to ensure a consistent quality of YMRE were performed by Jeongwoo Pharmaceutical Company's guidelines and specification including HPLC fingerprints (supplementary content, Figures  S1 and S2 in Supplementary Material available online at https://doi.org/10.1155/2017/9814836). In brief, the ethanol extract of 16 medicinal materials was prepared in the ratio described in YMR composition (see details in supplementary content). The ethanol extract was then evaporated in a 50°C water bath under vacuum until ethanol was completely removed. A total of 310.7 g of the final extract was obtained from 2 kg of mixture of 16 medicinal materials, and thus production rate was 15.54%. This final extract (YMRE) was weighed, resuspended in sterile distilled water, and orally administered to mice at a dose of 100 mg/kg/day. Sixteen medicinal plants listed in either KP (Korean Pharmacopeia) or KHP (Korean Herbal Pharmacopeia) were used to make YMRE, and the specification of each medicinal plant was established in accordance with the requirements described in KP or KHP. The official information for all plants in YMRE was provided in supplementary content.

### 2.3. Noise Exposure and ABR Test

The animals were unrestrained and placed below the horns of two loudspeakers: a low frequency woofer and a high frequency tweeter. Electrical Gaussian noise (33120A, Agilent) was delivered to the speakers for 3 h after power amplification to induce hearing loss. The noise level was adjusted to 100 dB and monitored using a 1/4-inch microphone linked to a sound level meter (Tucker-Davis Technologies, FL, USA). In order to record ABR, the animal was anesthetized with a mixture of xylazine (60 mg/kg, i.p.) and ketamine (60 mg/kg, i.p.), and body temperature was maintained at 37.5–38°C with a thermostatic heating pad. Three subdermal needle electrodes (ear tips; 3.5 mm, Nicolet Biomedical, Inc.) were used to record the ABR thresholds measured at 4, 8, 16, and 32 kHz. At each frequency, the test was performed in a descending sequence from 90 dB HL in 10-dB steps until the ABR response disappeared. The noninverting electrode was inserted at the vertex, and the reference and grounding electrodes were placed on the two earlobes. Hearing thresholds were determined before administration of YMRE and exposure to noise, and on days 1, 5, and 10 after the noise stimulation. Hardware and software from Tucker-Davis Technology (TDT system III, FL, USA) were used for stimulus generation and biosignal acquisition.

### 2.4. Hair Cell Examination

After functional hearing test, cochleae of Balb/C mice were quickly removed from the temporal bone, fixed in 4% paraformaldehyde in phosphate-buffered saline (PBS) at 4°C for 12 h, and washed with cold PBS 3 times. Cochleae were incubated in DECAL (10% ethylenediaminetetraacetic acid [EDTA]) solution at 4°C for 12 h which was repeated for 3 days. For hair cell counting, the cochlear basilar membrane containing the organ of Corti was carefully dissected out and stained with fluorophore-conjugated phalloidin (Sigma P1915) diluted in PBS (1 : 5000) for 30 min in the dark at room temperature. After being washed with PBS 3 times, samples were mounted on glass slides in glycerin, coverslipped, and examined under a confocal microscope (IX81, Olympus, Japan). The number of outer hair cells in the basal turn of the cochlea from each sample was counted.

### 2.5. Real-Time Polymerase Chain Reaction

Total RNA from cochlear tissue collected from each mice group (*n* = 5) on days 1 and 5 after with/without-noise exposure was extracted using Trizol (FATRR001; Favorgen) according to manufacturer's instructions. Cochlear RNA samples were then subjected to gene expression analysis. One microgram of RNA was used to synthesize cDNA, which was then subjected to quantitative polymerase chain reaction (qPCR; Illumina) using SYBR mix (Roche) and the following primers: glyceraldehyde 3-phosphate dehydrogenase (forward 5′-GATCATCAGCAATGCCTCCT-3′ and reverse 5′-TGTGGTCATGAGTCCTTCCA-3′), TNF*α* (forward 5′-CTGAGGTCAATCTGCCCAAGTAC-3′ and reverse 5′-CTTCACAGAGCAATGACTCCAAAG-3′), IL-1*β* (forward 5′-TCTTTGAAGTTGACGGACCC-3′ and reverse 5′-TGAGTGATACTGCCTGCCTG-3′), and HO-1 (forward 5′-CCCACCAAGTTCAAACAGCTC-3′ and reverse 5′-AGGAAGGGGGTCTTAGCCTC-3′).

### 2.6. Hematoxylin and Eosin (H&E) Staining

H&E staining was initially performed on liver and kidney samples that had been formalin-fixed and paraffin-embedded. In brief, paraffin was removed using xylene followed by ethyl alcohol dehydration. The sections were rehydrated with tap water and stained with Gill hematoxylin for 4 min. After another tap-water wash, specimens were irrigated with Scott tap water for 40 s. After an ethyl alcohol rinse, the eosin-phloxine stain was applied for 35 s. The sections were then dehydrated in ethyl alcohol, cleared with xylene, and coverslipped using a Vectashield® mounting medium (Burlingame, CA).

### 2.7. Cryosection

Cochleae were decalcified as described in Section 2.4 and washed with PBS. Samples were further incubated in 30% sucrose for 12 h at 4°C and repeated for an additional day. Samples were then transferred into a mixture of 30% sucrose and OCT compound (1 : 1) and kept for 12 h at 4°C. After being slowly frozen in butanol containing liquid nitrogen, the samples were cryosectioned at 10 microns, placed on a glass slide, and dried at room temperature for 1-2 h. The sectioned cochlea tissues were used for TUNEL assay and 4HNE staining.

### 2.8. TUNEL Assay for DNA Fragmentation

Cryosections of cochlear tissue were subjected to terminal deoxynucleotidyl transferase (TdT) dUTP nick-end labeling (TUNEL) assay (Roche, #11684817910) to detect the fragmentation of nuclear DNA, and labeling procedure was performed according to the manufacturer's instructions with slight modification. Briefly, the cochleae removed from each mouse group were fixed in solution (pH 7.5) containing 4% buffered formalin and 0.1% glutaraldehyde (15714S, Electron Microscopy Sciences) at 4°C for 24 h. Samples were then permeabilized in proteinase K solution for 10 min at room temperature. They were washed once with washing buffer provided by the manufacturer and incubated in the freshly prepared DNA-labeling solution containing TdT enzyme and BrdUTP for 16 h at room temperature. After being washed twice with rinse buffer, tissues were stained with the red fluorescence-labeled anti-BrdU antibody at room temperature for 1 h, followed by counterstaining with Vectashield with DAPI.

### 2.9. 4-Hydroxy-2-nonenal (4HNE) Staining

Isolated cochleae fixed following the protocol above were decalcified in 10% EDTA at 4°C for 3 days and incubated in 30% sucrose solution. Samples were then embedded in Tissue-Tek OCT compound (Sakura, #4538) for cryosection. Tissue sections were incubated in 3% bovine serum albumin (BSA; Bioworld) solution for 30 min blocking, followed by further incubation with anti-4HNE (Abcam, #ab46545) and secondary antibody. After washing, mounting medium was added to the slides and samples were analyzed under the confocal microscope (IX81, Olympus, Japan) with a digital camera (DP70, Olympus).

### 2.10. Statistical Analysis

Each experiment was performed at least three times, and the results were represented as mean ± SD. Statistical significance was calculated with one-way analysis of variance (ANOVA; Tukey's post hoc analysis) and accepted at the level of *p* < 0.05.

## 3. Results

### 3.1. YMRE Attenuates Noise-Induced Hearing Loss in Mice

**Figure 1:**
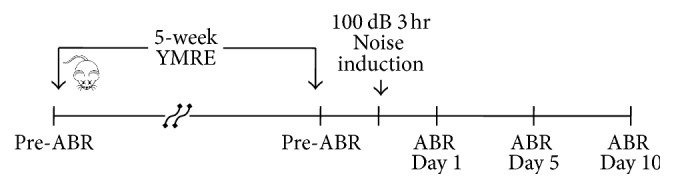
*Experimental scheme of animal study*. Mice were divided into four different groups after initial ABR was measured: Cont, noise, YMRE, and YMRE + noise. For groups with YMRE treatment, mice were administered orally with a dose of 100 mg/kg/day for 5 weeks, while sterile DW (s-DW) was given to mice groups that were not treated with YMRE. After 5 weeks of treatment with either s-DW or YMRE, ABR was measured prior to and on days 1, 5, and, 10 after exposure to 100 dB noise (YMRE: Yang Mi Ryung extract, ABR: auditory brainstem response).

**Figure 2:**
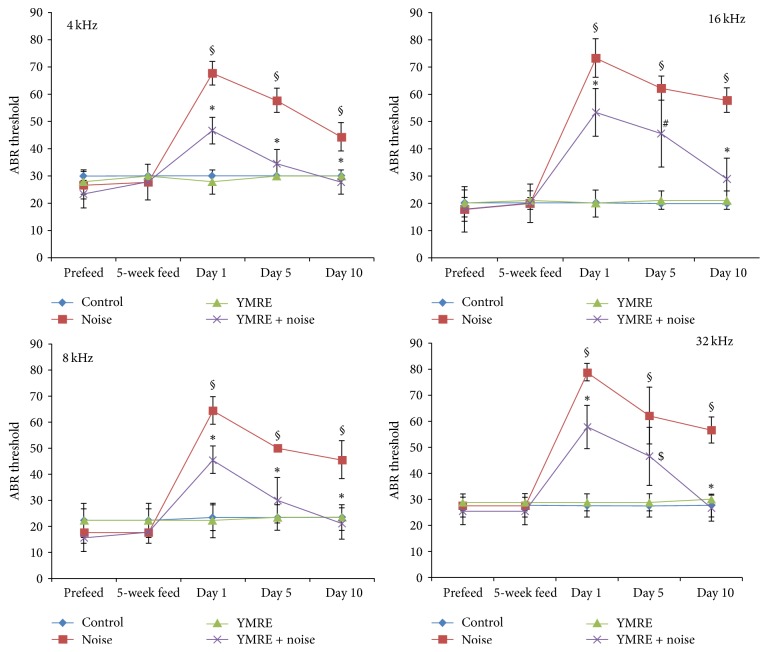
*Inhibition by YMRE of noise-induced hearing loss in mice*. Hearing impairment was determined by ABR thresholds in the mice of control group, noise group, YMRE group, and YMRE + noise group. ABR thresholds were determined at 4, 8, 16, and 32 kHz in all four groups. ^§^*p* < 0.0001 versus “control” group. ^*∗*^*p* < 0.0001 versus “noise” group. ^#^*p* < 0.002 versus “noise” group. ^$^*p* < 0.009 versus “noise” group. The mean values of ABR recordings from the left ear of 9 mice in each group are shown. The results were represented as mean ± SD.

Our group has been interested in developing the preventive interventions to palliate hearing loss induced by noise stimulation and tested whether YMRE has the otoprotective effect on NIHL in mice. As outlined in Figure 1, hearing thresholds were measured at the beginning of the experiment, prior to noise exposure, and on days 1, 5, and 10 after the noise and compared among treatment groups. The ABR thresholds were measured at 4, 8, 16, and 32 kHz, and the test was performed at each frequency in a descending sequence from 90 dB HL in 10-dB steps until the ABR response disappeared. As shown in Figure 2, exposure to high level noise, 100 dB, for 3 h, induced a remarkable threshold shift in the first 24 h after noise. The average hearing thresholds in noise-exposed mice ranged from 65 to 75 dB, showing a 40–50 dB shift when compared to that in the control group on day 1. Even though a gradual recovery of ABR in noise group was observed in a time dependent manner, it was very limited. At all postexposure time points, noise-exposed animals kept a significant threshold shifts ranging from 17 to 40 dB, compared to unexposed mice. However, the mean thresholds in mice pretreated with YMRE ranged from 45 to 55 dB on day 1 after noise stimulation, and threshold shifts were at least 20 dB lower than those in noise-only group at all time points tested. Moreover, when measured on day 10 after noise stimulation, hearing ability of YMRE pretreatment group noticeably was recovered to a level almost comparable to that of the control group, and such protection was observed at all frequencies tested. In contrast, the noise group without pretreatment of YMRE on day 10 continued to show a 20–40 dB shift in hearing thresholds compared to the control group. These data strongly suggest that pretreatment of YMRE could be a promising approach in protecting hair cells from NIHL.

### 3.2. Preadministration of YMRE Protects against Noise-Induced Cytotoxicity in Organ of Corti

**Figure 3:**
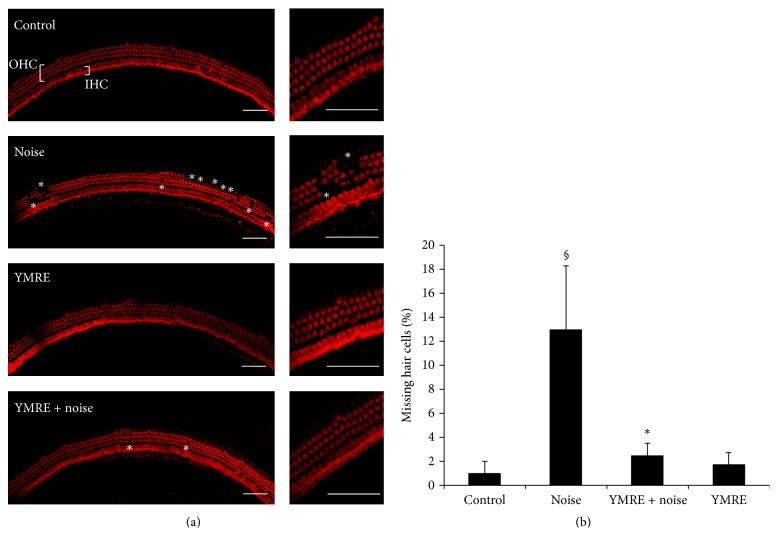
*Immunofluorescence phalloidin staining of the organ Corti*. (a-b) Hair cells were stained with fluorophore-conjugated phalloidin and observed using a confocal microscope (IX81, Olympus). Immunostaining shows the loss of outer hair cells in the basal turn of cochleae exposed to noise (noise) and the protective effects observed in mice administered with YMRE for 5 weeks (YMRE + noise). (a) Asterisks indicate the areas of the misalignment and hair cell loss. The data are the representative results obtained from three independent experiments. Scale bar 50 *μ*m. Right panels show the representative areas expanded from pictures in the left of each group. (b) The quantification of outer hair cells missing in four cochlear explant groups. The results were represented as mean ± SD, *n* = 5. ^§^*p* < 0.001 versus “control” group. ^*∗*^*p* < 0.001 versus “noise” group.

Since noise exposure has been known to damage cochlear hair cells, we next examined whether or not YMRE prevents noise-induced loss of hair cells in mice. The organ of Corti was carefully isolated from mice pretreated with YMRE for 5 consecutive weeks prior to noise stimulation, and the loss of outer hair cells in the basal turn of cochleae was then compared to that of the control or noise stimulation groups by analyzing phalloidin staining of organ of Corti. As shown in Figure 3(a), no hair cell loss or mild loss in hair cells was observed in control group, while noise stimulation induced substantial damage to the arrangement of three outer rows of hair cells and resulted in noticeable hair cell loss. However, the misalignment and hair cell loss observed in noise stimulation group were markedly reduced in the mice group preadministrated with YMRE prior to noise stimulation. This suggests that YMRE plays a pivotal role in preventing ototoxicity induced by noise. We also counted the number of missing hair cells in each group, and the result was shown in Figure 3(b). Our data demonstrated that about 14% hair cells were missing in the noise stimulation group, while there were no significant changes in numbers of missing cells in both YMRE + noise and YMRE only group, compared to control group (Figure 3(b)). These results suggest that YMRE might have antiapoptotic properties against noise-induced cell death.

### 3.3. Apoptotic Cell Death Caused by Noise Was Blocked by YMRE in Mice Cochleae

**Figure 4:**
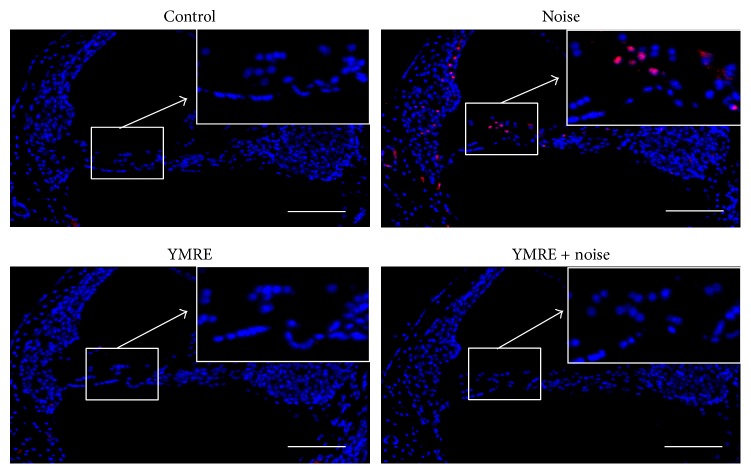
*YMRE protects mouse cochlea from apoptotic cell death caused by noise*. TUNEL assay was used to detect apoptotic cell death (red) in the basal turn of cochlea collected 10 days after noise stimulation with or without preadministration of YMRE for 5 consecutive weeks. Each panel is a representative image of each experimental group (4 slides per animal, *n* = 5). Scale bar: 100 *μ*m. All slides from “noise” group animals show positive staining.

Accumulating research has reported that exposure to intense noise causes hair cell death mainly through apoptosis, resulting in activation of caspases and triggering of cytochrome c release in cochlea [17–19]. This prompted us to evaluate whether the protective effect of YMRE involved inhibition of noise-induced apoptotic cell death. Since DNA fragmentation is a characteristic hallmark of apoptosis, we used the detection method that incorporates fluorescently modified nucleotide at the 3′-OH ends of fragmented DNA. Therefore, TUNEL staining assay was used to determine apoptotic cells after the noise stimulation either with or without YMRE pretreatment. As shown in Figure 4, exposure to noise markedly increased TUNEL-positive (red) cells in the organ of Corti, while there was no positive staining detected in cells from YMRE + noise and YMRE only groups which were very similar to the control group. These data demonstrate that YMRE noticeably protected cells from apoptotic cell death caused by noise stimulation.

### 3.4. YMRE Greatly Suppressed ROS Levels Elevated by Noise in Organ of Corti

Studies have revealed that reactive oxygen species (ROS) play a pivotal role in several apoptotic cell death pathways in mouse auditory tissues, and that ROS is considered as a major cause of many types of sensorineural hearing loss, including NIHL [5, 7]. Furthermore, many research groups found that ROS levels were commonly increased in noise-mediated hearing loss [7, 20, 21]. ROS, highly reactive molecules, not only causes DNA damage and strand breaks, but also leads to protein oxidation or lipid peroxidation. Thus, 4HNE, the product of lipid peroxidation, has been a useful indicator of ROS and a common hallmark in the assessment of oxidative stress levels [22, 23].

**Figure 5:**
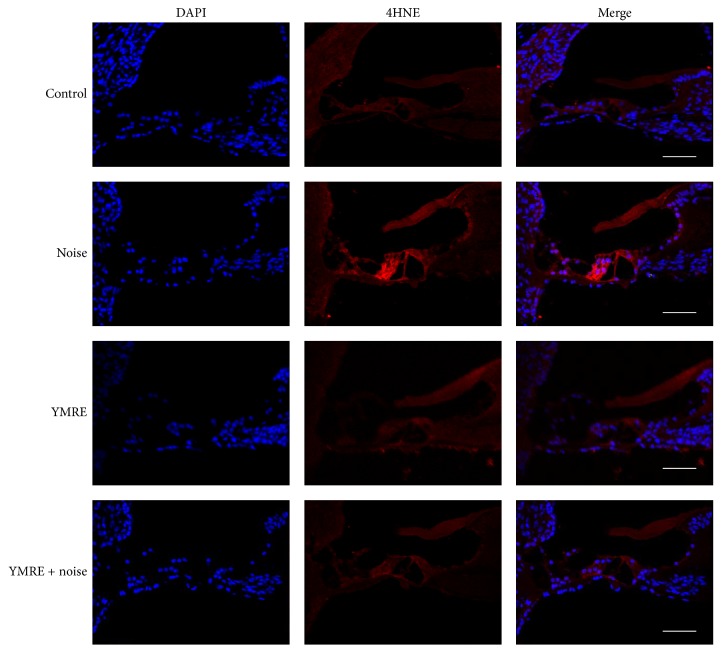
*YMRE suppresses the induction of ROS by noise in mouse cochlea*. Immunofluorescence signals against 4HNE, an indicator of ROS production, were measured in the basal turn of cochlea collected 5 days after noise stimulation with or without pretreatment of YMRE for 5 consecutive weeks. Each panel is a representative image of each experimental group (4 slides per animal, *n* = 5). Scale bar: 50 *μ*m. All slides from “noise” group animals show positive staining.

To test whether the ability of YMRE to inhibit noise-mediated apoptosis resulted from regulating ROS production, we measured the levels of 4HNE by immunostaining analysis. The sections of cochleae isolated 5 days after noise stimulation were stained with anti-4HNE antibody (red) and blue fluorescence is DAPI, indicating nuclei. Our results showed that exposure to noise significantly increased the 4HNE levels in mice cochleae. In contrast, very weak staining of 4HNE was shown throughout the cochleae in the YMRE + noise group as well as the saline-treated control and YMRE only groups (Figure 5). When comparing the noise group to the YMRE + noise group, noise stimulation failed to elevate ROS levels in mice groups preadministered consecutively for 5 weeks with YMRE, demonstrating that YMRE could be a promising interventional approach to protect cells from oxidative stress induced by noise. Taken together, our data suggest that YMRE protects mice from NIHL by eliminating cytotoxic ROS in cochleae possibly through activation of scavenging systems, thereby preventing subsequent cell death.

### 3.5. Inflammatory Responses Were Suppressed by YMRE Preadministration in Organ of Corti

ROS has been associated with the inflammatory responses, and emerging evidence demonstrated that inflammation might be a central contributor in acoustic cochlear damage [6, 24–26]. Since our data showed the inhibitory effect of YMRE on hair cell loss and 4HNE expression induced by noise exposure, we further studied if YMRE regulates levels of inflammatory cytokines and antioxidant enzymes in the organ of Corti. Total RNAs extracted from the organ of Corti were subjected to qPCR to measure the expression levels of both pro- and anti-inflammatory genes.

Accumulating evidence reported that HO-1, an antioxidant enzyme, may be a primary protective effector against noise-induced ototoxicity [27]. Acting as one of early-responsive genes induced by oxidative stress such as noise stimulation, HO-1 has also been induced by the treatment with antioxidants and fortifies the adaptive responses to oxidative stress and apoptotic cell death [28, 29]. Therefore, we hypothesized that the protective effect of YMRE against acoustic trauma might occur through the further induction of HO-1. However, we were not able to see any significant changes in HO-1 expression at both days 1 and 5 in the current experimental setting, except for 1.8-fold induction by noise only on day 1, compared to that in control.

**Figure 6:**
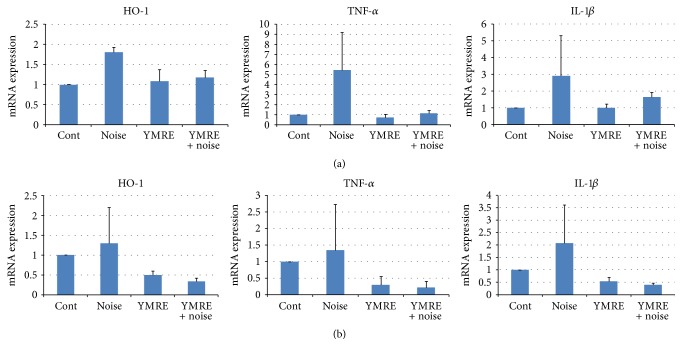
*Reduction of inflammatory cytokines induced by noise in mouse cochlea by YMRE*. The mRNA expression levels of HO-1, TNF-*α*, and IL-1*β* were measured by quantitative polymerase chain reaction (qPCR), and the means ± SD (the fold changes over the control group) were presented from three independent experiments. Cochlea samples were collected on day 1 (a) or day 5 (b) after exposure to 100 dB of noise and subjected to qPCR analysis. The results are represented as mean ± SD, *n* = 5.

We also evaluated the expression level of several key inflammatory markers. Among those genes tested, mRNA levels of TNF*α*, and IL-1*β* were induced at one day after noise treatment by 5- and 3-fold, respectively, and TNF*α* level was reduced to the similar expression level of control group on day 5 after noise. Such induction of TNF*α* and IL-1*β* levels in noise group seemed to be downregulated in YMRE + noise group, suggesting that YMRE may have a protective effect against noise-induced inflammation. Such protective effect was also observed even on day 5 (Figure 6(b)) after noise exposure, although to a lesser extent than that on day 1 (Figure 6(a)). In addition, YMRE itself did not have any influence on expression levels of those genes.

Our data suggested that YMRE may protect hair cells from noise-induced damage by suppressing the expressions of inflammatory genes such as TNF*α* and IL-1*β*.

### 3.6. YMRE Administration Does Not Cause Any Signs of Hepatotoxicity and Nephrotoxicity in Mice

**Figure 7:**
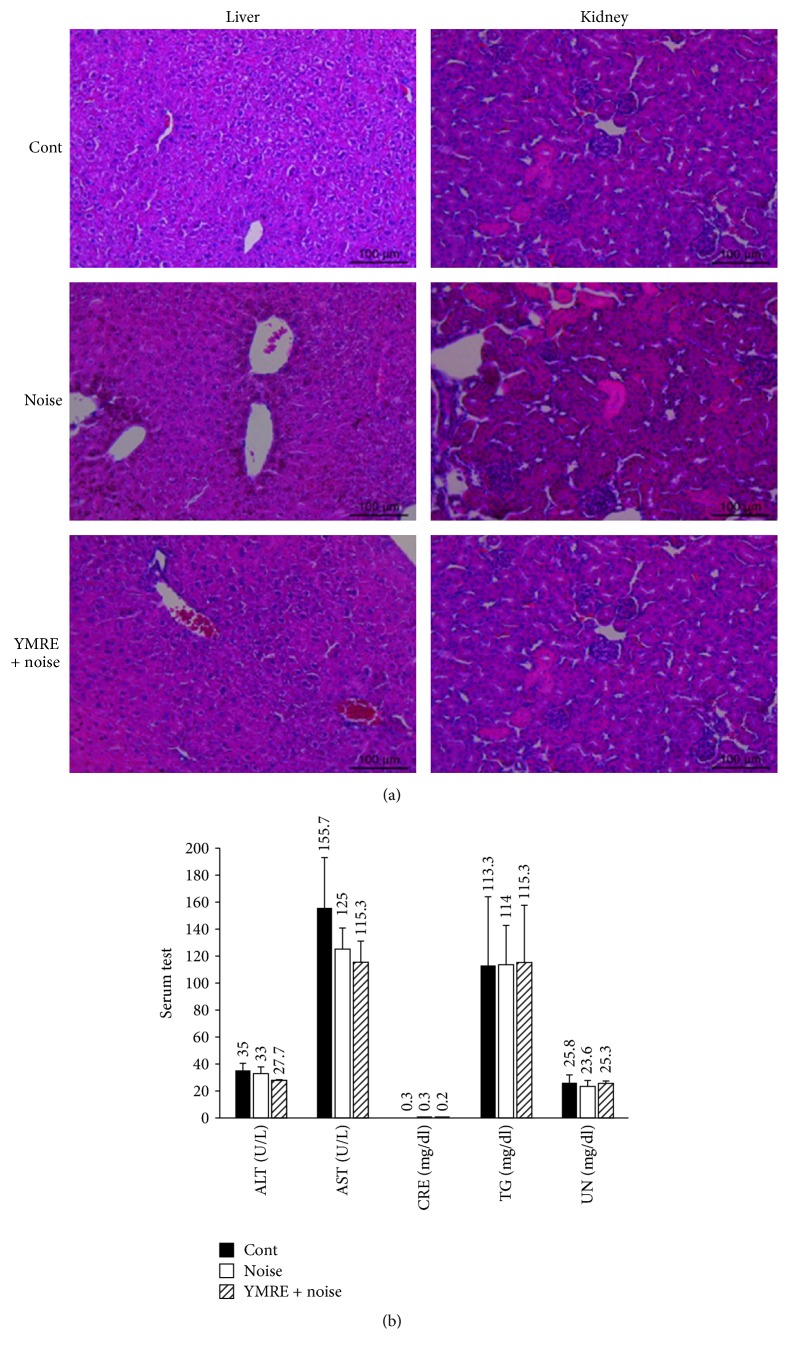
Histological and hematological evaluation after administration of YMRE. (a) H&E staining. Photomicrograph (200x) of liver and kidney sections after administration of YMRE (100 mg/kg/day, 5 weeks) followed by exposure to noise (YMRE + noise) or noise only without administration of YMRE (noise), compared to control (Cont). Mice were sacrificed 5 days after noise stimulation and blood, liver, and kidney were collected for analysis. Scale bar: 100 *μ*m. (b) Serum levels of ALT (alanine transaminase), AST (aspartate transaminase), CRE (creatinine), TG (triglyceride), and UN (blood urea nitrogen) were determined. The results are represented as mean ± SD, *n* = 5.

Orally administered drugs are absorbed in the gastrointestinal (GI) tract and subsequently distributed to many different parts of the body and metabolized. Liver and kidney are the most important organs for metabolism and elimination of biologically active substances, but they are also frequent targets for toxic components and metabolites. Therefore, it is important to assess safety and toxicity in the animal system to validate the use of biologically active substances and determine possible drug candidates. Samples of blood, liver, and kidney were collected 5 days after noise exposure, and biochemical parameters such as alanine aminotransferase (ALT), aspartate aminotransferase (AST), creatinine (CRE), triglyceride (TG), and blood urea nitrogen (UN) were evaluated using serum. Histological changes in liver and kidney sections were determined by H&E staining. As shown in Figure 7(a), liver sections of mice in the control group showed normal hepatic structure and hepatocytes with prominent nuclei, portal area, and central vein, and there were no signs of inflammation or degenerative changes observed. There were also no differences found in the liver sections of the noise group. Moreover, the general structure of liver lobules was clear and normal without any pathological changes in YMRE treated groups. Similarly, kidney sections in YMRE preadministered groups as well as in control and noise groups showed no noticeable pathological changes such as inflammation or necrosis. The results of serum parameters of liver and kidney were displayed in Figure 7(b), and we were unable to detect any significant differences in all the parameters including ALT, AST, CRE, TG, and UN in all mice groups in this study. Levels of serum parameters tested were all within a normal range and unaltered by noise or YMRE treatment. Our data suggest that YMRE is a very safe drug that does not cause any sign of hepatotoxicity or nephrotoxicity in mice.

## 4. Discussion and Conclusions

Hair cells are primary sensory receptor cells that detect sound, at the beginning of the hearing process. Initially, researchers believed that damage to hair cells was caused by the pure force of vibrations from loud sounds. However, recent studies have demonstrated that exposure to harmful noise triggers the formation of molecules, such as ROS, and induction of inflammatory genes inside the ear, resulting in damage or death of the hair cells [27, 30]. These cells are extremely delicate and sensitive and do not regenerate once damaged or lost. Furthermore, there are no known therapeutic treatments to restore hearing. We have searched for possible drug candidates to protect hair cells from ototoxic stimuli and thus prevent hearing loss. Among those candidates, the present study demonstrates YMRE as a new preventive invention against NIHL. The pretreatment with YMRE prior to noise exposure not only reduced ABR thresholds induced by noise, but also restored hearing ability to that of normal control, while the noise-only group did not fully recover the shift in ARB thresholds. Moreover, our results provide convincing evidence that the protective effect of YMRE against NIHL occurs through blocking ROS levels and inflammatory gene expression in cochleae.

Accumulating studies have supported the oxidative stress and inflammation as the leading causes of cochlear damage in NIHL [7]. It has been shown that larger quantities of ROS were produced by increased aerobic respiration of mitochondria following noise stimulation, which caused substantial loss of sensory hair cells through apoptosis and necrosis [31]. Therefore, antioxidants such as N-acetylcysteine, coenzyme Q10, and several vitamins have been reported to reduce noise-induced ototoxicity by inhibiting oxidative stress in animal models [32].

To date, various inflammatory proteins and genes have been implicated in the cochlear inflammation following noise exposure [6, 33]. Such key factors include chemokine (C-C motif) ligand 2 (CCL2), cyclooxygenase-2 (Cox-2), TNF*α*, IL-1*β*, and intercellular adhesion molecule-1 (ICAM-1), all of which have been important players to recruit and infiltrate inflammatory cells into cochlea after noise stimulation in animal model [34, 35]. In this study, mRNA expression levels of TNF*α* and IL-1*β* induced by noise exposure seem to be suppressed by YMRE pretreatment prior to noise, even though we were not able to show statistical significance for qPCR data. The occurrence of high error bar in noise group in Figure 6 might be due to the individual variances in response, the number (*n* = 5 per group) of animals used in this study, or time points to collect the tissue samples for RNA, since we collected tissues for RNA isolation on days 1 and 5 after noise. It might be necessary to test the expression levels of inflammatory genes at earlier time points, for example, less than 12 h. At this moment, we do not know which inflammatory genes and proteins would mediate the protective effect of YMRE against NIHL and the exact molecular mechanism by which YMRE suppresses cochlear inflammation and protects hair cells from noise-induced damage. Based on our data in Figures 5 and 6, we could carefully suggest that ROS, some inflammatory molecules including TNF*α* and IL-1*β*, and antioxidant enzymes such as HO-1 might be targets for YMRE for its protection against NIHL. In support of the destructive role of inflammation in cochlea, recent studies demonstrated that the pharmacological inhibition of Cox-2 and TNF*α* prevents permanent threshold shift following noise overexposure [36, 37].

Vasoconstriction in cochlea has been reported as an important contributor to hair cell damage, and emerging evidence suggested that inflammatory genes including TNF*α* reduced systemic and/or regional blood flow in inner ear, leading to NIHL [3]. Intense noise has been known to induce vasoconstriction by forming the lipid peroxidation product, 8-iso-PGF2*α*, in the cochlea, and the reversal of cochlear blood flow reduced by noise exposure with a specific antagonist against 8-iso-PGF2*α* offered protection for hearing ability [38, 39]. Because YMR has been known to improve blood flow and used for the treatment of circulatory disorders, we speculate that YMRE protects from NIHL by possibly increasing or improving blood flow in the inner ear. However, the molecular mechanism behind this hypothesis needs to be further investigated.

In conclusion, while further preclinical or clinical studies using other in vivo hearing loss models are required to confirm the hearing protection of YMRE, outcomes of this study add a new possible therapeutic utility of YMRE, suggesting its clinical intervention against hearing loss.

## Supplementary Material

Figure S1. HPLC fingerprints of YMRE acquired at 254 nm. YMRE extract were analyzed by HPLC and chromatograms of the sample was recorded for 90 min. Fourteen common peaks were detected at 254 nm. The retention time and retention area of these 14 peaks were shown. Figure S2. HPLC fingerprints of YMRE acquired at 365 nm. YMRE extract were analyzed by HPLC and chromatograms of the sample was recorded for 90 min. Twenty seven common peaks were detected at 365 nm. The retention time and retention area of these 27 peaks were shown.

## Abbreviations

NIHL: Noise-induced hearing loss
ABR: Auditory brainstem response
YMRE: Yang Mi Ryung extract
ROS: Reactive oxygen species
4-HNE: 4-Hydroxy-2-nonenal.
