# Supplementary material for: Protective Effect of Yang Mi Ryung® Extract on Noise-Induced Hearing Loss in Mice
Source: Evid Based Complement Alternat Med. 2017 Nov 15;2017:9814836. doi: 10.1155/2017/9814836 (PMC5705878; doi:10.1155/2017/9814836)
Supplement: Supplementary file 1 — Figure S1. HPLC fingerprints of YMRE acquired at 254 nm. YMRE extract were analyzed by HPLC and chromatograms of the sample was recorded for 90 min. Fourteen common peaks were detected at 254 nm. The retention time and retention area of these 14 peaks were shown. Figure S2. HPLC fingerprints of YMRE acquired at 365 nm. YMRE extract were analyzed by HPLC and chromatograms of the sample was recorded for 90 min. Twenty seven common peaks were detected at 365 nm. The retention time and retention area of these 27 peaks were shown. [file 9814836.f1.zip › Supplementary content.docx]

**Supplementary Content**

1. **Yang Mi Ryung^®^ (YMR, Jeongwoo Pharmaceutical Co., Ltd) Composition**

Two kilogram of mixture of 16 medicinal materials includes *Cyperus Rhizome* 330 g, *Peony Root* 168 g, *Corydalis Tuber* (KP) 61 g, *Anethi Fructus* (KHP) 33 g, *Glycyrrhizae Radix* (KP) 61 g, *Rehmanniae Radix Preparata* (KP) 168 g, *Atractylodis Rhizoma Alba* (KP) 132 g, *Cinnamomi Cortex* (KP) 61 g, *Moutan Radicis Cortex* (KP) 61 g, *Cnidii Rhizom*a (KP) 132 g, *Amomi Fructus* (KP) 99 g, *Zingiberis Rhizoma* (KP) 61 g, *Angelicae Gigantis Radix* (KP) 168 g, *Evodiae Fructus* (KP) 61 g, *Citrus Unshiu Peel* (KP) 168 g, and *Poria Sclerotium* (KP) 168 g. These medicinal plants used were all listed in either KP (Korean Pharmacopeia) or KHP (Korean Herbal Pharmacopeia), and the specification of each medicinal plant was established in accordance with the requirements described in KP or KHP.

1. Cyperus Rhizome (KP) : Cyperus Rhizome is the rhizome of *Cyperus rotundus* Linné (Cyperaceae), from which rootlets have been removed.
2. Peony Root (KP) : Peony Root is the root of *Peonia lactiflora* Pallas or allied plants (Paeoniaceae).
3. Corydalis Tuber (KP) : Corydalis Tuber is the tuber of *Corydalis ternata* Nakai or *Corydalis yanhusuo* W.T.Wang (Papaveraceae).
4. Anethi Fructus (KHP) : Anethi Fructus is the fruit of *Anethum graveolens* Linné (Umbelliferae).
5. Licorice (Glycyrrhizae Radix et Rhizoma (KP)) : Licorice is the root and rhizome with or without the periderm, of *Glycyrrhiza uralensis* Fisher, *Glycyrrhiza glabra* Linné or *Glycyrrhiza inflate* Batal. (Leguminosae).
6. Rehmanniae Radix Preparata (KP) : Prepared Rehmannia Root is the root of *Rehmannia glutinosa* Liboschitz ex Steudel (Scrophulariaceae), with the application of steaming.
7. Atractylodis Rhizoma Alba (KP) :Atractylodes Rhizome White is the rhizome, with or without periderm, of *Atractylodes japonica* Koidzumi or *Atractylodes macrocephala* Koidzumi (Compositae), or from which the periderm has been removed.
8. Cinnamomi Cortex (KP) : Cinnamon Bark is the bark of the trunk of *Cinnamomum cassia* Presl or other species of the same genus (Lauraceae), or such bark from which a part of the periderm has been removed.
9. Moutan Radicis Cortex (KP) : Moutan Root Bark is the root bark of *Paeonia suffruticosa* Andrews (Paeoniaceae).
10. Cnidii Rhizoma (KP) : Cnidium Rhizome is the rhizome or the rhizome passed through hot water of *Cnidium officinale* Makino or *Ligusticum chuanxiong* Hort. (Umbelliferae).
11. Amomi Fructus (KP) :Amomum Fruit is the ripe fruit or seed mass of *Amomum villosum* Lourerio var. *xanthioides* T.L.Wu et Senjen and *Amomum villosum* Lourerio (Zingiberaceae).
12. Zingiberis Rhizoma (KP) : Ginger is the dried rhizome of *Zingiber officinale* Ros-coe (Zingiberacece).
13. Angelicae Gigantis Radix (KP) : Angelica Gigas Root is the root of *Angelica gigas* Nakai (Umbelliferae).
14. Evodiae Fructus (KP) : Evodia Fruit is the fruit of *Evodia rutaecarpa* Bentham, *Evodia rutaecarpa* Bentham var. *officinalis* Huang or *Evodia rutaecarpa* Bentham var. *bodinieri* Huang (Rutaceae).
15. Citrus Unshiu Peel (KP) : Citri Unshius Pericarpium */* Citrus Unshiu Peel is the ripe pericarp of *Citrus unshiu* Markovich or *Citrus reticulate* Blanco (Rutaceae).
16. Poria Sclerotium (KP): Poria is the sclerotium of *Poria cocos* Wolf (Polyporaceae).
17. **Prepartion of Yang Mi Ryung^®^ Extract (YMRE)**

Two kilogram of 16 medicinal materials was extracted with 20 liters of ethanol. The ethanol extract was then evaporated in a 50 ℃ water bath under vacuum until ethanol was completely removed. A total of 310.7 g of the final extract was obtained from 2 kg of mixture of 16 medicinal materials, and thus production rate was 15.54%.

1. **Supplementary Materials & Methods**

*High-performance liquid chromatography (HPLC) analysis*

HPLC analysis was performed on an YL-9100 series HPLC instrument equipped with a sample injector and a PDA detector (YoungLin, Republic of Korea). For HPLC analysis, a Gemini 5μm NX-C18 110Å (4.6 mm × 250 mm; Phenomenex Inc., USA) column was used as the stationary phase, and the injection volume was 20 μL. The HPLC sample was prepared to 1 g YMRE soluble in 100 mL methanol with 10 minutes sonicating. The mobile phase was composed of water (contained 0.1% formic acid) (A) and acetonitrile (B), with a gradient elution method: 0─10 min, a linear gradient from 10%B to 20%B; 10─30 min, a linear gradient from 20%B to 35%B; 30─45 min, a linear gradient from 35%B to 50%B; 45─75 min, a linear gradient from 50%B to 80%B; 75─90 min, a linear gradient from 80%B to 10%B. Flow rate was 0.7mL/min, and the peaks were detected at 254 and 365 nm. For identifying peak, peaks were ignored under the 300 mV.s in 254 nm and 50 mV.s in 365 nm.
