# Supplementary material for: Protective Effect of Yang Mi Ryung® Extract on Noise-Induced Hearing Loss in Mice
Source: Evid Based Complement Alternat Med. 2017 Nov 15;2017:9814836. doi: 10.1155/2017/9814836 (PMC5705878; doi:10.1155/2017/9814836)
Supplement: Supplementary file 1 — Figure S1. HPLC fingerprints of YMRE acquired at 254 nm. YMRE extract were analyzed by HPLC and chromatograms of the sample was recorded for 90 min. Fourteen common peaks were detected at 254 nm. The retention time and retention area of these 14 peaks were shown. Figure S2. HPLC fingerprints of YMRE acquired at 365 nm. YMRE extract were analyzed by HPLC and chromatograms of the sample was recorded for 90 min. Twenty seven common peaks were detected at 365 nm. The retention time and retention area of these 27 peaks were shown. [file 9814836.f1.zip › Supplementary Figure S1 and S2.docx]

**
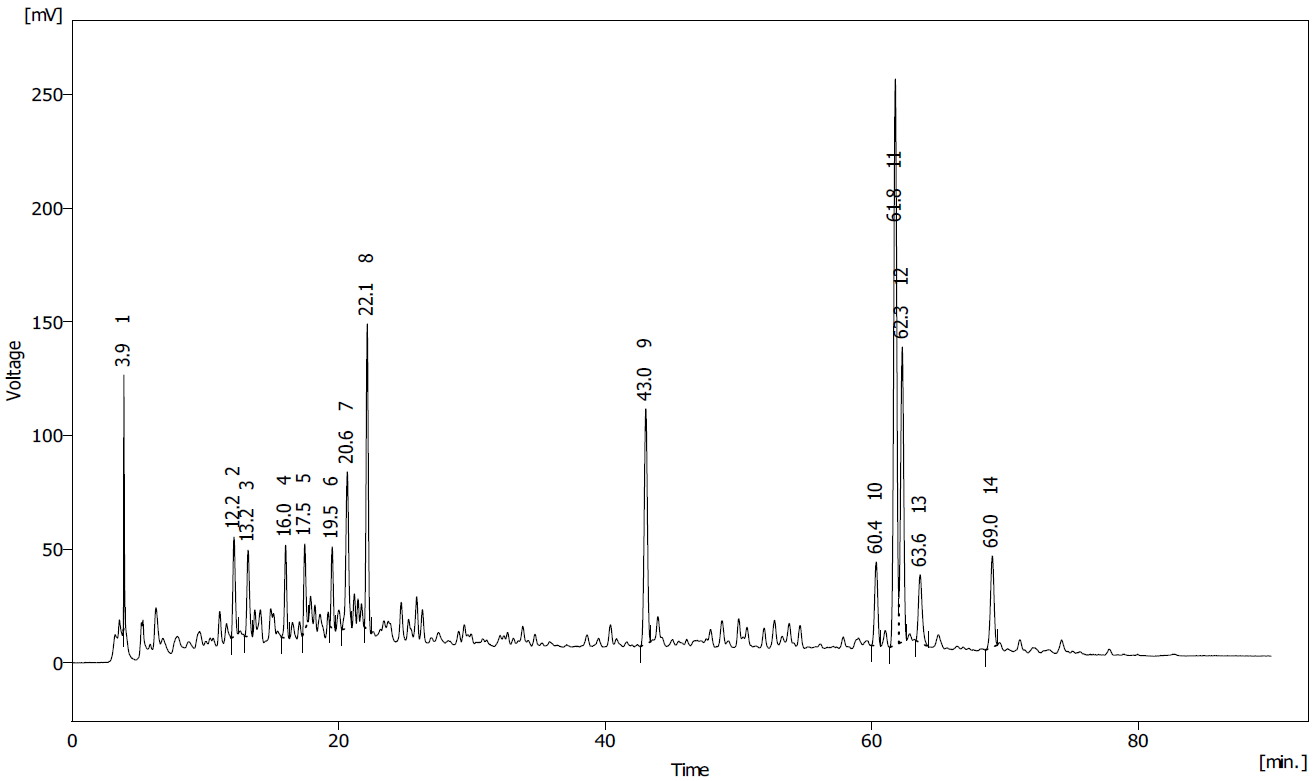
**


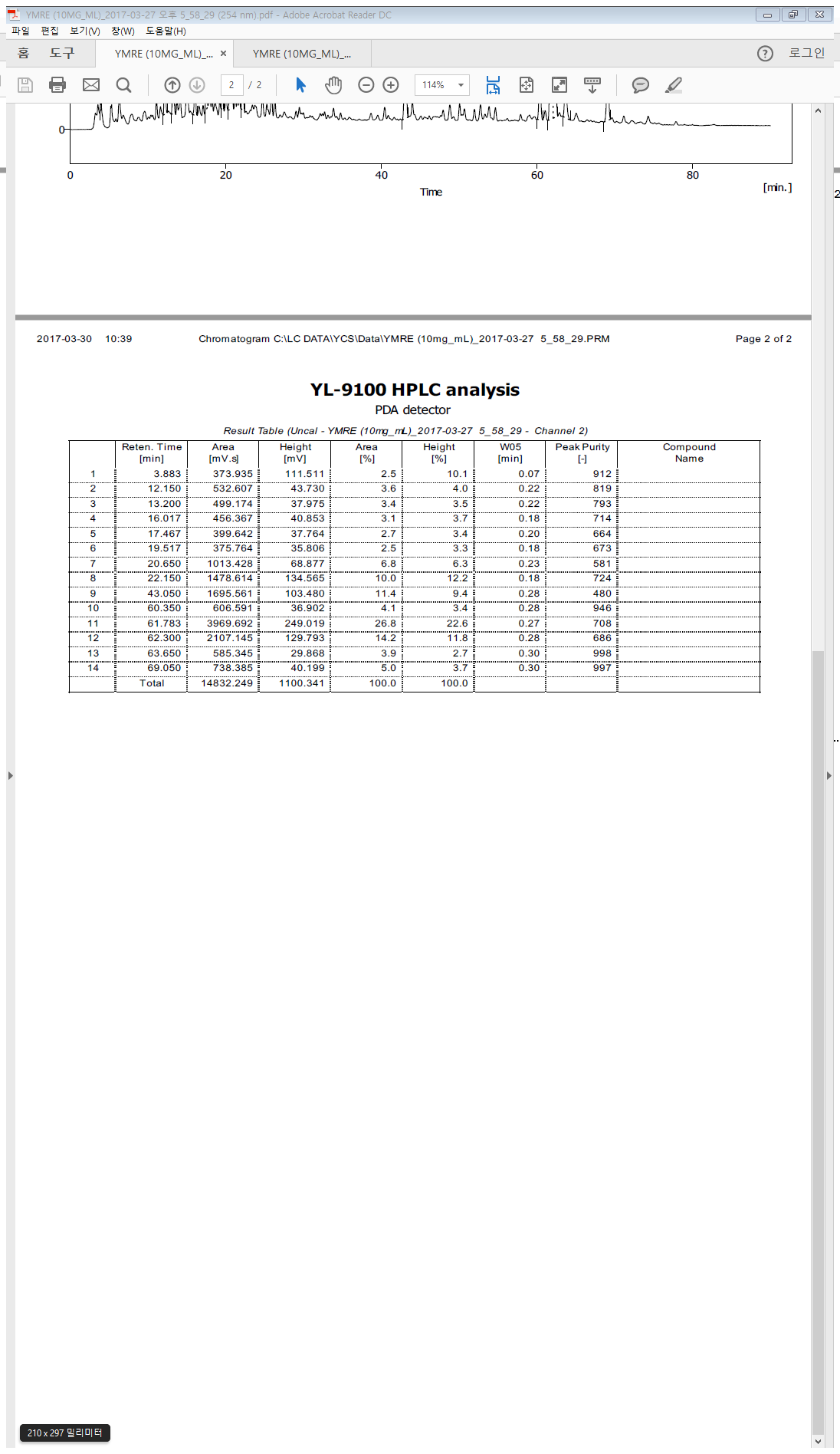


**Figure S1.** HPLC fingerprints of YMRE acquired at 254 nm

YMRE extract were analyzed by HPLC and chromatograms of the sample was recorded for 90 min. Fourteen common peaks were detected at 254 nm. The retention time and retention area of these 14 peaks were shown.

**
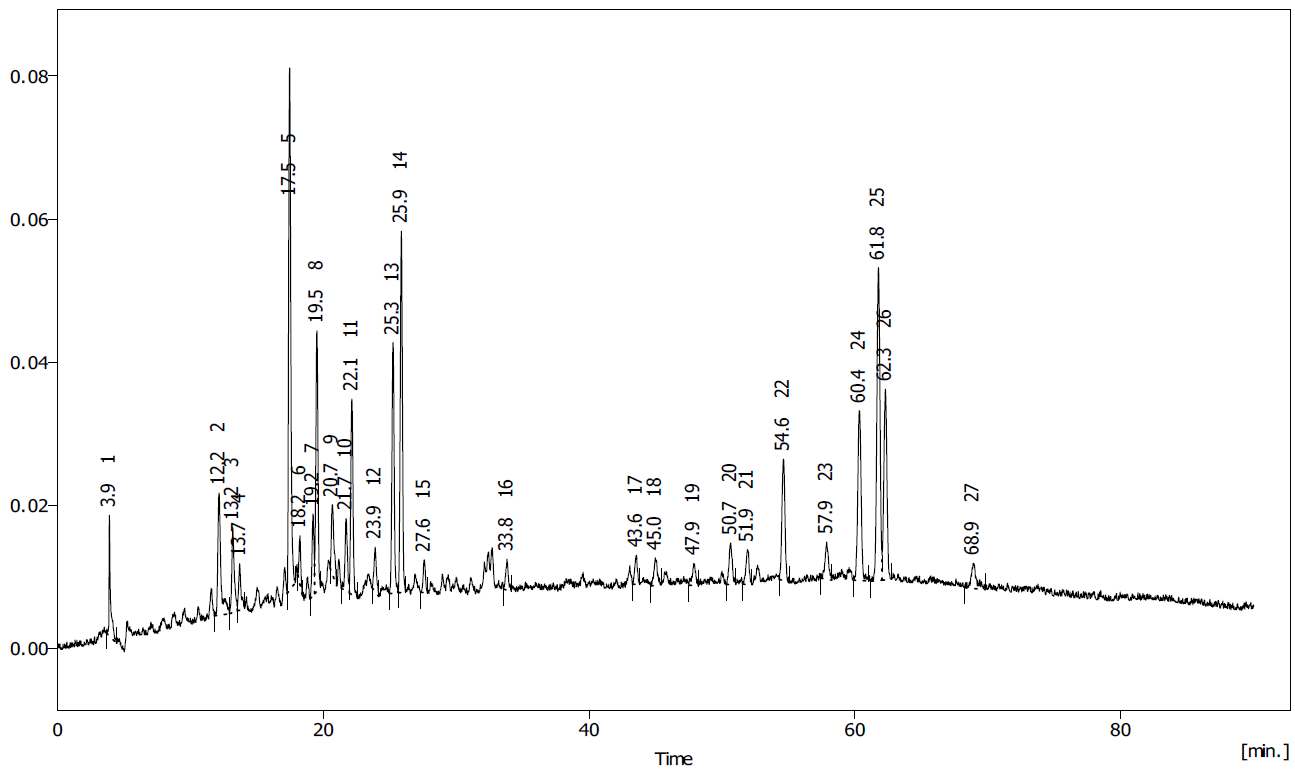
**

**
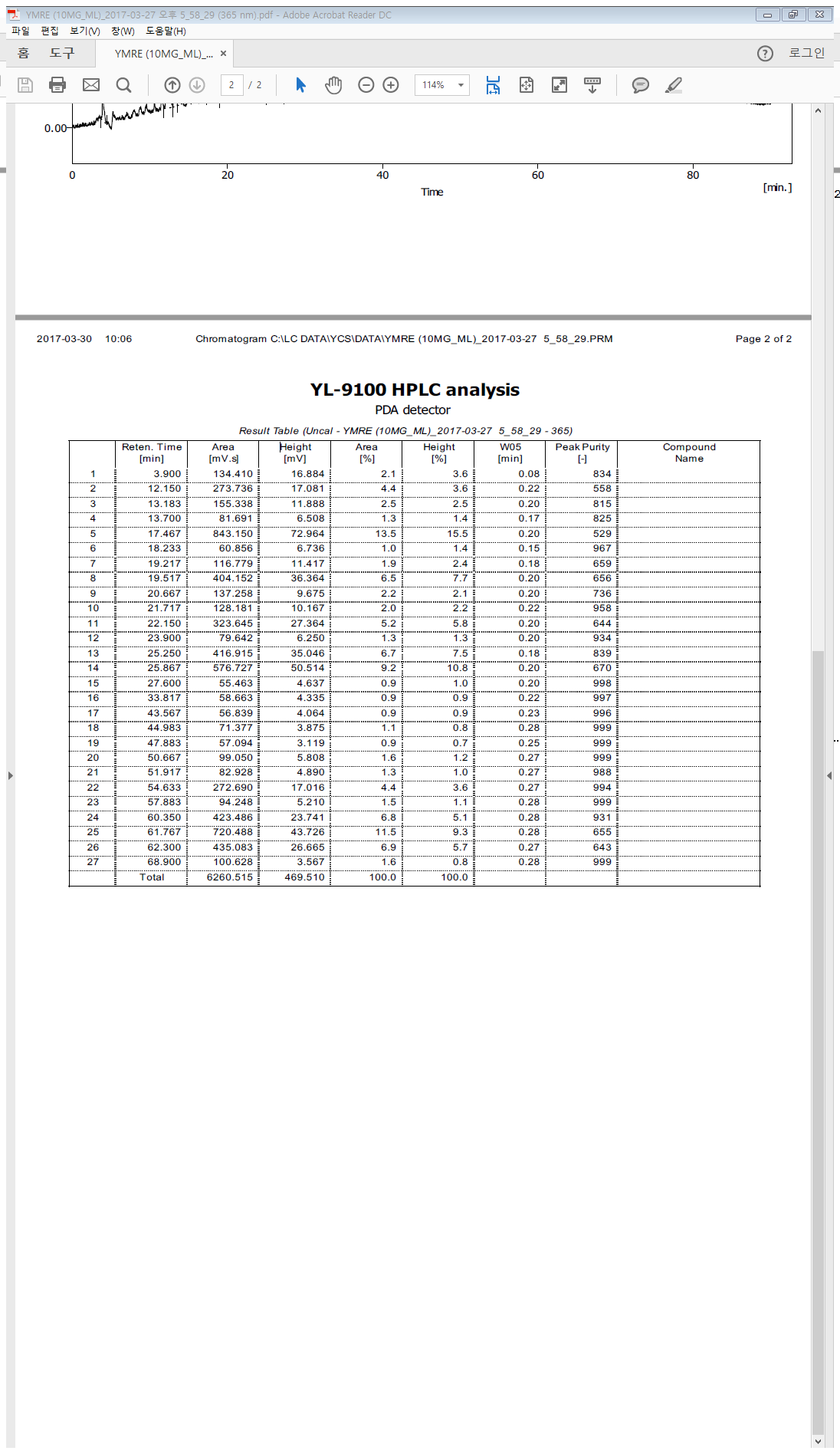
**

**Figure S2.** HPLC fingerprints of YMRE acquired at 365 nm

YMRE extract were analyzed by HPLC and chromatograms of the sample was recorded for 90 min. Twenty seven common peaks were detected at 365 nm. The retention time and retention area of these 27 peaks were shown.
